# DNA Methylation Profiles of the Brain-Derived Neurotrophic Factor (BDNF) Gene as a Potent Diagnostic Biomarker in Major Depression

**DOI:** 10.1371/journal.pone.0023881

**Published:** 2011-08-30

**Authors:** Manabu Fuchikami, Shigeru Morinobu, Masahiro Segawa, Yasumasa Okamoto, Shigeto Yamawaki, Norio Ozaki, Takeshi Inoue, Ichiro Kusumi, Tsukasa Koyama, Kounosuke Tsuchiyama, Takeshi Terao

**Affiliations:** 1 Division of Frontier Medicine, Department of Psychiatry and Neurosciences, Graduate School of Medical Sciences, Hiroshima University, Hiroshima, Japan; 2 Department of Psychiatry and Molecular Psychiatry, Nagoya University Graduate School of Medicine, Nagoya, Japan; 3 Department of Psychiatry, Hokkaido University Graduate School of Medicine, Sapporo, Japan; 4 Department of Neuropsychiatry, Oita University Faculty of Medicine, Yufu City, Oita, Japan; Chiba University Center for Forensic Mental Health, Japan

## Abstract

Major depression, because of its recurring and life-threatening nature, is one of the top 10 diseases for global disease burden. Major depression is still diagnosed on the basis of clinical symptoms in patients. The search for specific biological markers is of great importance to advance the method of diagnosis for depression. We examined the methylation profile of 2 CpG islands (I and IV) at the promoters of the brain-derived neurotrophic factor (BDNF) gene, which is well known to be involved in the pathophysiology of depression. We analyzed genomic DNA from peripheral blood of 20 Japanese patients with major depression and 18 healthy controls to identify an appropriate epigenetic biomarker to aid in the establishment of an objective system for the diagnosis of depression. Methylation rates at each CpG unit was measured using a MassArray® system (SEQUENOM), and 2-dimensional hierarchical clustering analyses were undertaken to determine the validity of these methylation profiles as a diagnostic biomarker. Analyses of the dendrogram from methylation profiles of CpG I, but not IV, demonstrated that classification of healthy controls and patients at the first branch completely matched the clinical diagnosis. Despite the small number of subjects, our results indicate that classification based on the DNA methylation profiles of CpG I of the BDNF gene may be a valuable diagnostic biomarker for major depression.

## Introduction

Major depression was among the 10 diseases with the greatest global burden in 2001 [1] and it is predicted to become the second leading causes of disability-adjusted life years in 2020 based on systematic analyses of population health data [2]. There are marked cross-national and cross-regional differences in the prevalence of major depression [3], [4], [5], [6], [7]. In addition, diagnostic differences are suggested to be associated with the varied proportion of patients receiving any specific mental health care [3]. Although the underlying reasons are not fully known, the difference in rates of major depression across countries could conceivably be due in part to social, economic, and cultural differences. On the other hand, the diagnostic system for major depression, which rely on assessment of patient symptoms, such as the Diagnostic and Statistical Manual of Mental Disorders fourth edition (DSM-IV), rather than an objective laboratory test, may also account for the difference in rates of major depression. Hence, the search for biological markers for major depression could be important for improving patient care and for the development of more effective drug treatments.

Although numerous studies have been undertaken to identify biomarkers in major depression, no biological markers proposed to date, including the dexamethasone suppression test (DST) [8], [9] and the combined dexamethasone/corticotrophin-releasing hormone (DEX/CRH) test [10], have been sufficiently specific to warrant inclusion as a diagnostic criterion [11]. Likewise, Brunoni et al. conducted a systematic review and meta-analysis of brain-derived neurotrophic factor (BDNF) levels in patients with major depression, which demonstrated a difference in blood BDNF levels between pre-treatment patients and healthy controls. In fact, since a number of studies showed decreased blood BDNF levels in patients with major depression [12], [13], it is possible that blood BDNF levels could serve as a potential biomarker for major depression. However, there is an evident overlap in the BDNF levels between these patients with depression and healthy controls. In this context, it would be of great interest to develop a highly sensitive diagnostic biomarker for major depression [14].

One of the most important requirements for a clinically useful biomarker is that it should be non-invasive. Towards that end, analyses of protein, RNA, and DNA levels from blood samples have been conducted by many researchers. Although mRNA and/or protein detection techniques can potentially be useful, the instability of these molecules leads to lack of reproducibility of test results and the need for normalization [15]. DNA-based analyses are more convenient due to the amplifiable and stable nature of DNA. Methylation of cytosine residues is in most cases chemically and biologically stable over time, and epigenetic changes are potentially reversible by treatment with pharmacological agents or by environmental stimuli, whereas genetic changes are irreversible [16]. Thus, great attention has been focused on the correlation between the hypermethylation of promoter-associated CpG islands and the transcriptional activity of genes, and the use of DNA methylation patterns as a biomarker in cancer and other complex or multifactorial diseases has been advocated [17], [18], [19].

Despite the promise of using DNA methylation as a biomarker, few studies to date have examined the possibility of using epigenetic biomarkers in psychiatric disorders [20]. Tsankova et al. recently demonstrated that social defeat stress, an animal models of depression, affects transcription of BDNF through changes in histone acetylation and DNA methylation in the rat hippocampus [21]. It is well known that the stress-induced decreases in BDNF and antidepressant-stimulated increases in BDNF play important roles in the pathophysiology and therapeutic mechanisms of depression, respectively. In addition, a different type of stimulus, depolarization, was also reported to upregulate BDNF via a decrease in CpG methyaltion at the promoter regions of the BDNF gene [22], [23].

Based on these findings, we examined the methylation profile of 2 CpG islands at the promoters of exon I and IV of the BDNF gene using genomic DNA from peripheral blood of Japanese patients with major depression and healthy controls to identify an appropriate epigenetic biomarker for the objective diagnosis of depression.

## Materials and Methods

### Subjects

Twenty patients with major depression and 18 healthy controls participated in this study. Demographic characteristics of the participants are shown in Table 1. All subjects were Japanese. All patients were diagnosed by trained psychiatrists according to DSM-IV criteria (American Psychiatric Association, 1994), on the basis of unstructured interviews and information from medical records, and through the use of a structured clinical interview, the Japanese version of the Mini-International Neuropsychiatric Interview [24], [25] by a research psychiatrist. The severity of depression was evaluated using the Hamilton Rating Scale for Depression (HAM-D). All patients were free of any current or past diagnoses of substance-related disorders. Healthy controls, free of any current or past psychiatric or physical diagnoses and any first-degree relatives with major depression, were recruited by advertisement. Blood samples were collected at Hiroshima University hospital, Nagoya University hospital, Hokkaido University hospital, and Oita University hospital. This study was approved by the Ethics Committee of the Hiroshima University School of Medicine, by the ethics committee of the Nagoya University School of Medicine, by the ethics committee of the Hokkaido University School of Medicine, and by the Oita University Faculty of Medicine ethics committee. All subjects received a description of this study and gave written informed consent.

**Table 1:** Demographic characteristics of subjects.

Group	Age (years: Mean ±S.D.)	HAM-D score (Mean ±S.D.)	Duration of untreated (Weeks: Mean ±S.D.)	Education (years: Mean ±S.D.)
Control (N = 18, 10M/8F)	42.3±9.6			13.78±2.05
Major depression (N = 20, 8M/12F, not medicated)	45.6±12.5	21.4±2.76	9.85±9.84	13.9±1.61

HAM-D: Hamilton Rating Scale for Depression.

### Selection of genomic regions of the BDNF gene for methylation analysis

**Figure 1:**
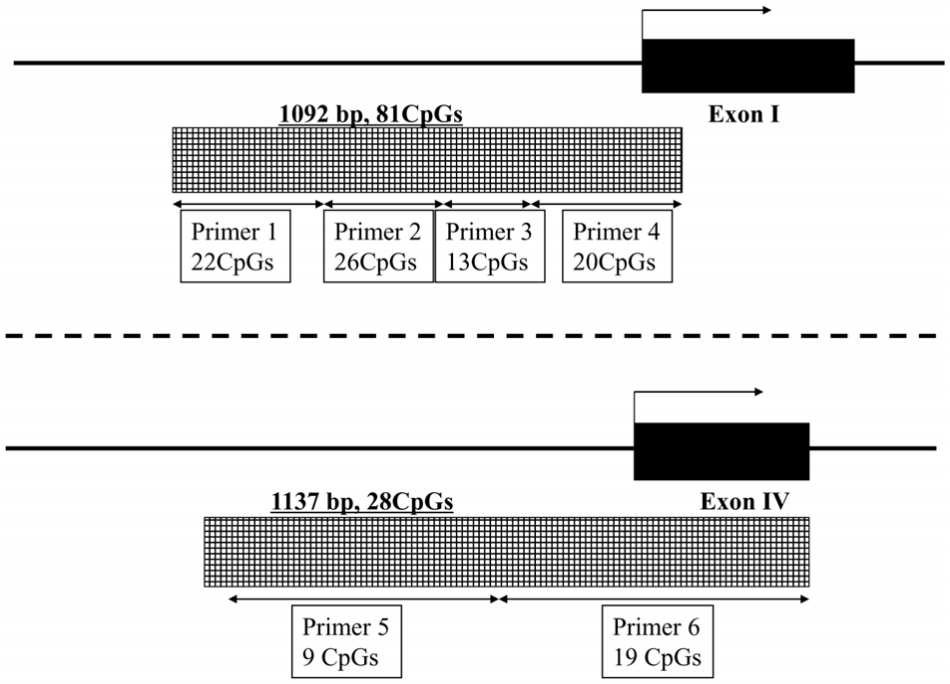
The schema of CpGs and primers used for DNA methylation analyses. The target region used for methylation analysis consists of 1092 bp including 81 CpGs upstream of exon I, and 1137 bp including 28 CpGs upstream of exon IV.

With respect to proximal promoter activity and preinitiation, the first exon of a gene is generally considered to be important in transcription [26], [27]. Alternatively, methylation of CpGs upstream of exon IV is proposed to be involved in the regulation of the BDNF gene under physical and pathological conditions [22], [28]. Thus, we chose the CpG island of the BDNF gene upstream of exon I (CpG I) and the cluster of CpGs at the upstream of exon IV (CpG IV) as targets for methylation analysis. The sequence of CpG I was identified by the use of the UCSC genome browser (http://genome.ucsc.edu/), (chr11:27743473–27744564 %GC = 60.5 and Obs/Exp CpG = 0.83). Since no CpG island was found based on the CpG island criteria of the UCSC genome browser (%GC>50, length >200 bp, Obs/Exp CpG>0.6) upstream of exon IV, we selected the area which was found to have proximal promoter activity (chr11:27722840–27723980) in previous experiments [23], [28]. Methylation primers were designed using Epidesigner software (http://www.epidesigner.com/) with the software's CpG island criteria (%GC>50, Obs/Exp CpG>0.6). The schemas of target regions of the BDNF gene used for methylation analysis and the primers used for PCR amplification are shown in Figure 1.

### DNA methylation analysis by MassARRAY

All blood samples were collected between 11:00 AM to 1:00 PM, prior to lunch. Blood samples (5 ml) were collected and placed in a vacuum tube containing heparin sodium and stored at −80°C. Genomic DNA was isolated using DNeasy® Blood & Tissue Kits (Qiagen, Hilden, Germany) according to the manufacturer's instructions. Genomic DNA (1 µg) was converted with sodium bisulfite using the EZ DNA methylation kit (Zymo Research, Orange, CA). The concentration of sodium bisulfite-treated DNA was measured using an ND-1000 spectrophotometer (NanoDrop Technologies, Inc., Wilmington, DE, USA), and 10 ng of treated DNA was applied in a region-specific PCR. The PCR reactions were carried out in a total volume of 5 µL using 1 µmol of each primer, 200 µM dNTP, 0.2 U HotStar Taq DNA polymerase (Qiagen), 15 mM MgCl2, and 10× PCR buffer (final concentration 1×). One of the two primers in the PCR amplification of the target regions is tagged with a T7 promoter sequence: cagtaatacgactcactatagggagaaggct. This includes ggg transcription start and an 8-bp insert (agaaggct) on the 5′ end. The reaction mix was preactivated for 4 min at 95°C. The reactions were amplified in 45 cycles of 95°C for 20 s, 56°C for 30 s, and 72°C for 60 s followed by 72°C for 3 min. Unincorporated dNTPs were dephosphorylated by adding 1.7 µL DNase free water and 0.3 U Shrimp Alkaline Phosphatase (SAP) (Sequenom, Inc., San Diego, CA, USA). The reaction was incubated at 37°C for 20 min and SAP was then heat inactivated for 5 min at 85°C. Subsequently, 2 µL of the PCR reaction were incubated for 3 h at 37°C with 5 µl of Transcleave mix (3.15 µl RNAse-free water, 0.89 µl 5×T7 Polymerase Buffer, 0.24 µl T Cleavage Mix, 0.22 µl 100 mM DTT, 0.44 µl T7 RNA&DNA Polymerase, 0.06 µl RNAse A (Sequenom) for concurrent in vitro transcription and base-specific cleavage. The resultant 10 to 20 nl cleavage reaction samples were spotted onto silicon matrix-preloaded chips (SpectroCHIP; SEQUENOM) using a MassARRAY nanodispenser (SEQUENOM), and analyzed using the MassARRAY Compact System matrix-assisted laser desorption/ionization-time-of-flight mass spectrometer (MALDI-TOF) (SEQUENOM). The spectra's methylation ratios were calculated using EpiTYPER software v1.0 (SEQUENOM). Triplicate independent analyses from sodium bisulfite-treated DNA sample were undertaken. The method yields quantitative results for each of the sequence-defined analytic units referred to as CpG units, which contain either 1 individual CpG site or an aggregate of downstream CpG sites. These methods divided 81 CpG sites in CpG I into 53 CpG units, and 28 CpG sites in CpG IV into 24 CpG units.

### Statistical analysis

Poor-quality and non-valuable data for the quantitative methylation of each CpG unit measured by MALDI-TOF-MS were excluded. CpG units that yielded data in greater than 25% of the samples passed initial quality control (QC). From these data, samples that yielded data for greater than 80% for all CpG units within an amplicon were met standard for inclusion in further analysis for that sample/amplicon pair. In subsequent analyses, CpG units for which data were available for less than 50% of all samples were excluded; samples which had data available for less than 50% of all CpG units were also excluded. These QC steps resulted in 35 available CpG units out of 53 CpG units in CpG I, and 19 available CpG units out of 24 CpG units in CpG IV.

The measurements after QC were combined in a data matrix, which was used in a 2-dimensional hierarchical clustering analysis with the ‘R’ software package for statistical computing (available at CRAN, http://cran.r=project.org/). Hierarchical clustering analyses were performed using hclust in the R cluster package, with Euclidean metric and complete linkage. Samples with closer methylation patterns are closely clustered.

We performed Peason's correlation coefficient test to examine the correlation between the methylation rate of each CpG units and the age of both patients and healthy controls or the total HAM-D scores in patients. The difference in the methylation rates of each CpG units between healthy controls and patients was analyzed by independent t-test. Significance was set at P<0.05.

## Results

The Raw Data from MassArray analysis indicating the methylation rates of each CpG units are shown as Data S1 and Data S2. Methylation rates of any CpG units had no statistically significant correlations with age or total HAM-D scores (Data S3).

### DNA methylation patterns of CpG I

**Figure 2:**
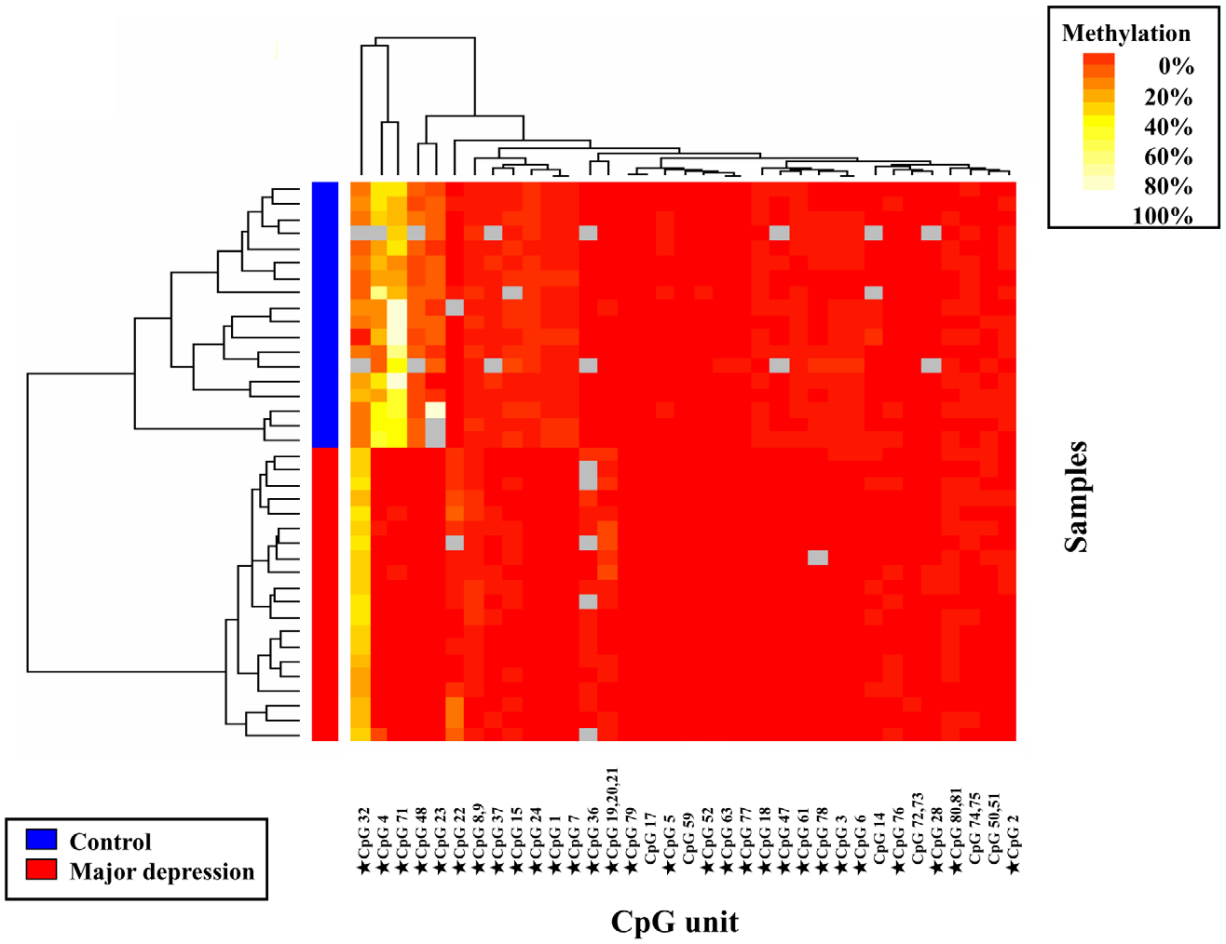
Hierarchic cluster analysis of subjects and their methylation profiles at CpG I of the BDNF gene. Two-way hierarchic cluster analysis of 38 samples (rows) and DNA methylation of CpG units at CpG I of the BDNF gene (columns). DNA methylation values are depicted by a pseudocolor scale as indicated (methylation increases from red [nonmethylated] to white [methylated]). Samples with overall poor data quality were removed before clustering. Gray denotes data of poor quality. Samples are color-coded according to the diagnoses of samples (legend depicted lower left). The stars(★) ahead of several CpG units indicate CpG units which have statistically significant p-values in subsequent analyses by t-test.

Two-way hierarchical clustering analysis of methylation rates of CpG units at CpG I was undertaken to classify samples and CpG units into clusters according to their similarity, and dendrograms were used to visualize the results. The DNA methylation profiles at BDNF CpG I of all subjects are shown in heat map format (Figure 2). The dendrogram acquired from clustering analysis is shown at left side of Figure 2. At the first branch of the dendrogram, we could distinguish between healthy controls and patients with major depression in complete concordance with classification based on clinical diagnosis. The height of the dendrogram indicates the similarity of subjects; the greater the height, the more similarity among divided subjects. Next, we analyzed the difference in methyration rates of each CpG units between healthy controls and patients with major depression (Table 2). The methylation rates of 29 CpG units out of 35 CpG units in BDNF CpG I were significantly different between these two groups.

**Table 2:** Results of independent t-test between healthy controls and patients of major depression.

	Control (mean±S.E.M)	Depression (mean±S.E.M)	t-value	P-value
CpG_1	12.4±0.47	2.1±0.27	19.6	8.5×10^−21^
CpG_2	7.29±0.34	5.0±0.42	4.14	1.98×10^−4^
CpG_3	8.26±0.58	3.25±0.47	6.75	7.04×10^−8^
CpG_4	58.85±4.12	2.4±1.19	13.76	6.58×10^−16^
CpG_5	4.69±0.53	0.65±0.28	6.88	4.67×10^−8^
CpG_6	8.26±0.58	3.25±0.47	6.75	7.04×10^−8^
CpG_7	12.44±0.47	2.1±0.27	19.6	8.5×10^−21^
CpG_8,9	12.88±0.28	10.9±0.69	2.56	0.015
CpG_14	5.22±0.93	4.25±0.77	0.81	0.422
CpG_15	14.25±0.94	5.85±0.54	7.96	1.89×10^−9^
CpG_17	0.22±0.13	0.9±0.37	−1.66	0.106
CpG_18	7.93±0.43	0.55±0.22	15.63	1.26×10^−17^
CpG_19,20,21	2.55±0.48	10.65±1.44	−5.12	1.04×10^−5^
CpG_22	3.89±0.66	15.69±2.57	−4.23	1.52×10^−4^
CpG_23	28.29±5.02	3.15±0.4	5.59	2.96×10^−6^
CpG_24	14.39±0.57	1.7±0.39	18.64	4.45×10^−20^
CpG_28	3.74±0.3	5.05±0.34	−2.85	7.12×10^−3^
CpG_32	36.77±2.23	62.3±1.4	−9.89	8.34×10^−12^
CpG_36	0.45±0.21	9.4±1	−8.34	6.24×10^−10^
CpG_37	11.5±0.37	5.5±0.35	11.76	6.85×10^−14^
CpG_47	6.08±0.4	3.25±0.22	6.45	1.75×10^−7^
CpG_48	27.68±0.88	1.55±0.29	29.61	7.14×10^−27^
CpG_50,51	6.3±0.26	5.5±0.42	1.57	0.126
CpG_52	4.26±0.26	1.5±0.22	8.06	1.43×10^−9^
CpG_59	2.4±0.29	2±0.26	1.04	0.305
CpG_61	8.81±0.41	3.05±0.34	10.99	4.73×10^−13^
CpG_63	3.69±0.43	2.15±0.23	3.23	2.6×10^−3^
CpG_71	74.27±4.18	3.45±0.43	17.77	2.12×10^−19^
CpG_72,73	3.43±0.33	3.65±0.36	−0.46	0.65
CpG_74,75	6.94±0.35	5.95±0.64	1.31	0.199
CpG_76	3.57±0.59	5.4±0.66	−2.04	0.049
CpG_77	3.69±0.43	2.15±0.23	3.23	2.7×10^−3^
CpG_78	7.53±0.75	2.58±0.34	6.22	3.54×10^−7^
CpG_79	0±0	0.004±0.17	−2.25	0.031
CpG_80,81	6.43±0.39	8.25±0.53	−2.74	9.6×10^−3^

The mean methylation rates among groups, t-value, P-value are shown. Significance was set at P<0.05.

### DNA methylation patterns of CpG IV

**Figure 3:**
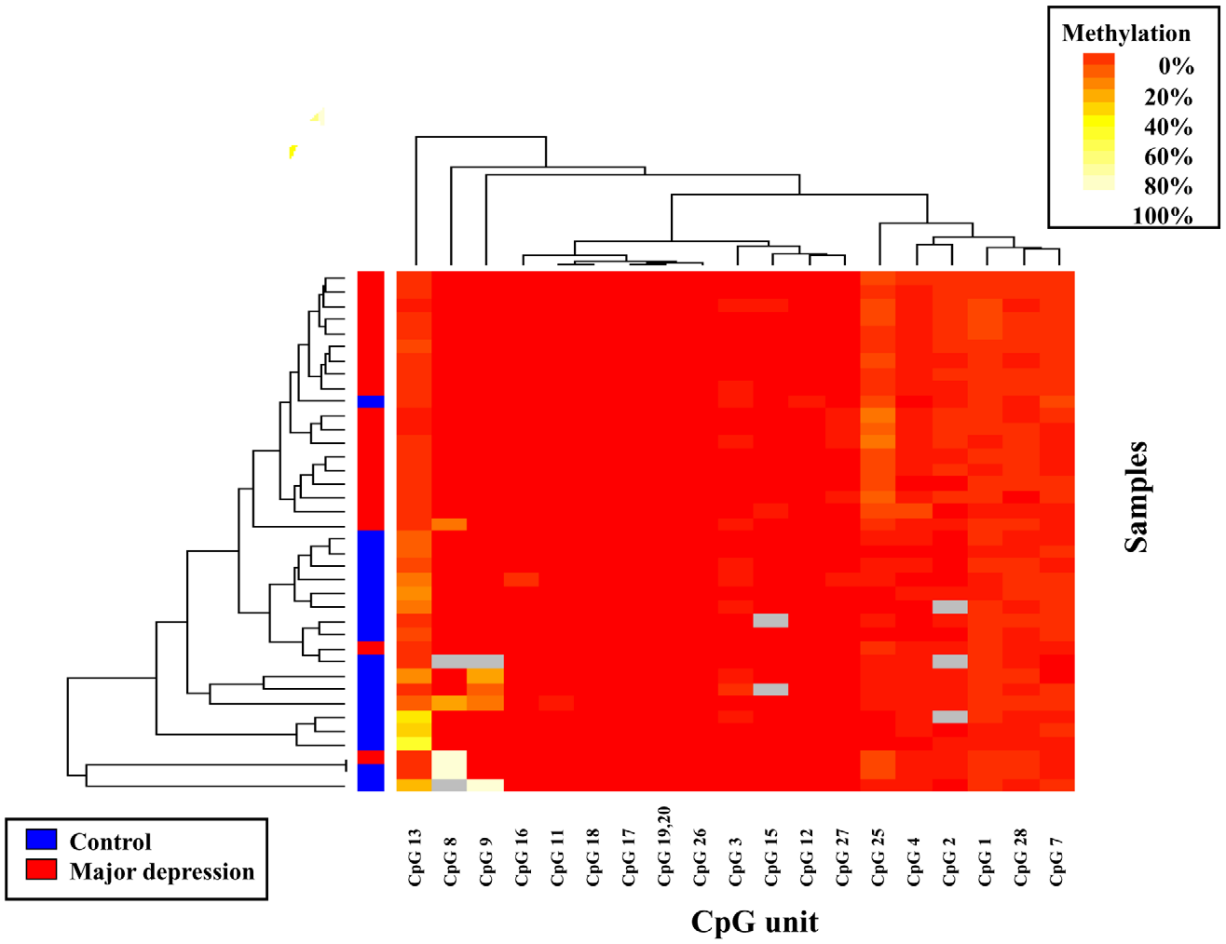
Hierarchic cluster analysis of subjects and their methylation profiles at CpG IV of the BDNF gene. Two-way hierarchic cluster analysis of 38 samples (rows) and DNA methylation of CpG units at CpG IV of the BDNF gene (columns). DNA methylation values are depicted by a pseudocolor scale as indicated (methylation increases from red [nonmethylated] to white [methylated]). Samples with overall poor data quality were removed before clustering. Gray denotes data of poor quality. Samples are color-coded according to the diagnoses of samples (legend depicted lower left).

Similar to the analysis of CpG I, we applied 2-way hierarchical clustering analysis of methylation rates of CpG units at CpG IV. The DNA methylation profiles at BDNF CpG IV of all subjects are shown in heat map format (Figure 3). The dendrogram acquired from clustering analysis is shown at left side of Figure 3. We were unable to distinguish subjects at any height in the dendrogram.

## Discussion

In the present study, we were able to accurately distinguish between patients with major depression and healthy controls, based on the methylation profiles of CpG units within CpG I, but not CpG IV, of the BDNF gene, and these results were completely concordant with clinical diagnoses. This finding indicates that quantitative methylation analysis within CpG I of the BDNF gene is helpful in the diagnosis of patients with major depression.

To our knowledge, this is the first study postulating the possibility of a DNA methylation marker in psychiatric disorders, though increased methylation of the promoter/exon IV of the BDNF gene was previously reported in Wernicke's area of the brain in suicide subjects [29]. Whereas Keller et al. demonstrated that a higher degree of methylation corresponded to lower BDNF mRNA in Wernicke's area, the influence of changes in DNA methylation profiles within CpG I on the transcription of the BDNF gene in blood are unknown. However, it is not necessary that DNA methylation markers for diseases always induces gene silencing [19].

Some limitations of the current study warrant mention. First, the sample size (n = 18 for control, n = 20 for major depression) in the current study is relatively small. Further studies using large samples are necessary for the clinical application in the future. Second, although numerous studies have demonstrated that the levels of gene expression in blood change after pharmacotherapy of psychiatric disorders [30], [31], it is uncertain whether the methylation profiles of DNA from peripheral blood in humans are affected by antidepressants. In this context, further studies examining the methylation profiles of CpG I in response to antidepressant treatment could reveal the influence of antidepressants on the DNA methylation, and subsequently identify whether the methylation profiles of CpG I from patients with major depression found in the current study are state or trait markers in major depression. Third, in contrast to SNPs and haplotypes [32], it is uncertain whether or not there are ethnic differences in DNA methylation profiles or not. Thus, these results may not apply to other races because of ethnic differences. Lastly, we evaluated the methylation profiles within only 2 CpG sites of the BDNF gene, but not genome-wide DNA methylation.

Based on MassARRAY analyses of the methylation profiles within the CpG island at the promoter of exon I of the BDNF gene in peripheral blood, we were able to accurately classify subjects into 2 groups (major depression, and healthy controls), and this classification was in good agreement with that obtained by clinical diagnosis. Thus, we propose that the DNA methylation profiles at CpG I of the BDNF gene could be a valid biomarker for the diagnosis of major depression.

## Supporting Information

Data S1 **Raw data from MassArray analysis of CpG I.**
(PPT)

Data S2 **Raw data from MassArray analysis of CpG IV.**
(PPT)

Data S3 **Correlation study between methylation rates of each CpG units and age or HAM-D score.**
(TIF)
