# Supplementary material for: DNA Methylation Profiles of the Brain-Derived Neurotrophic Factor (BDNF) Gene as a Potent Diagnostic Biomarker in Major Depression
Source: PLoS One. 2011 Aug 30;6(8):e23881. doi: 10.1371/journal.pone.0023881 (PMC3166055; doi:10.1371/journal.pone.0023881)

## Slide 1
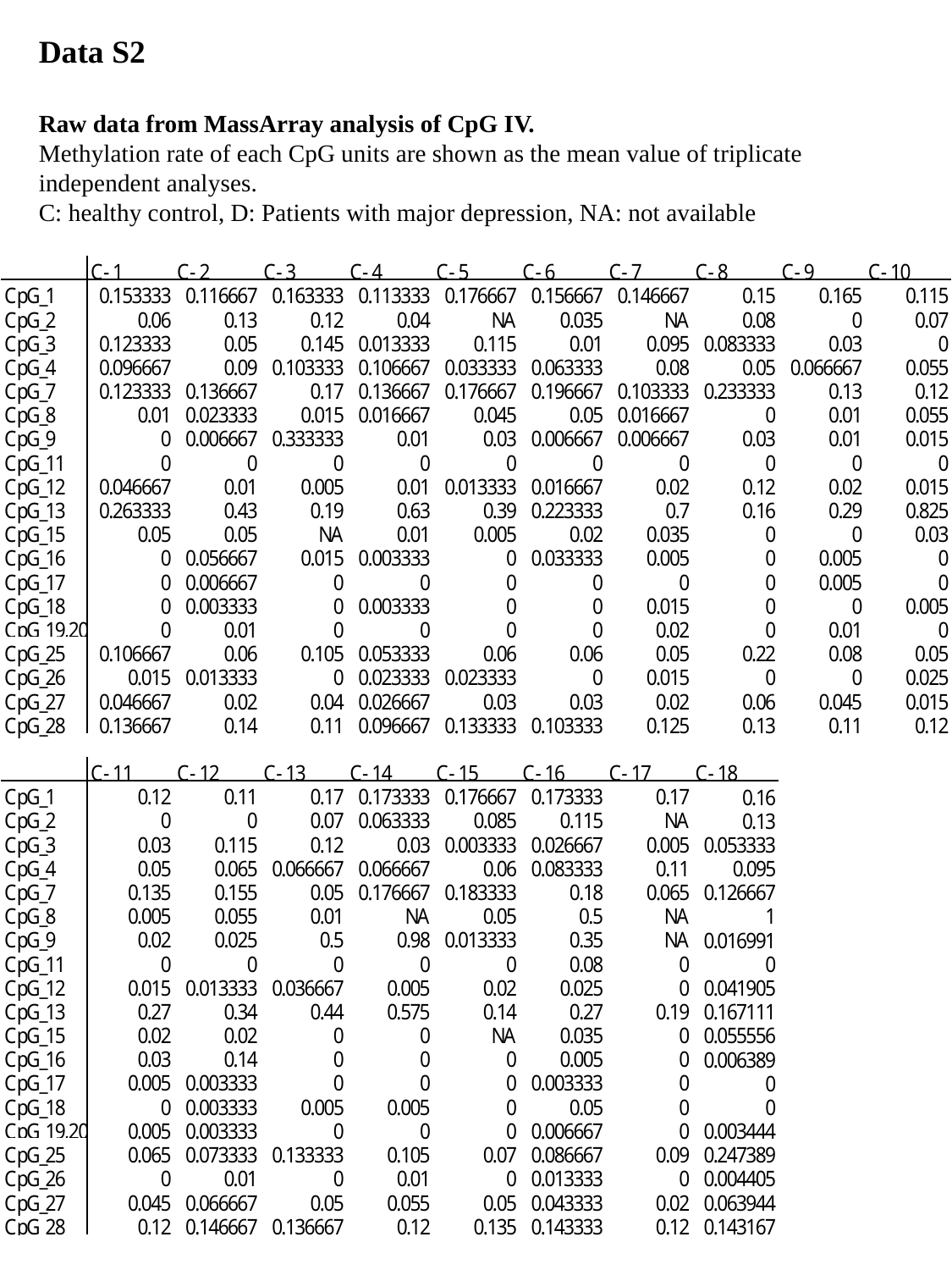

Data S2
Raw data from MassArray analysis of CpG IV.
Methylation rate of each CpG units are shown as the mean value of triplicate independent analyses.
C: healthy control, D: Patients with major depression, NA: not available

## Slide 2
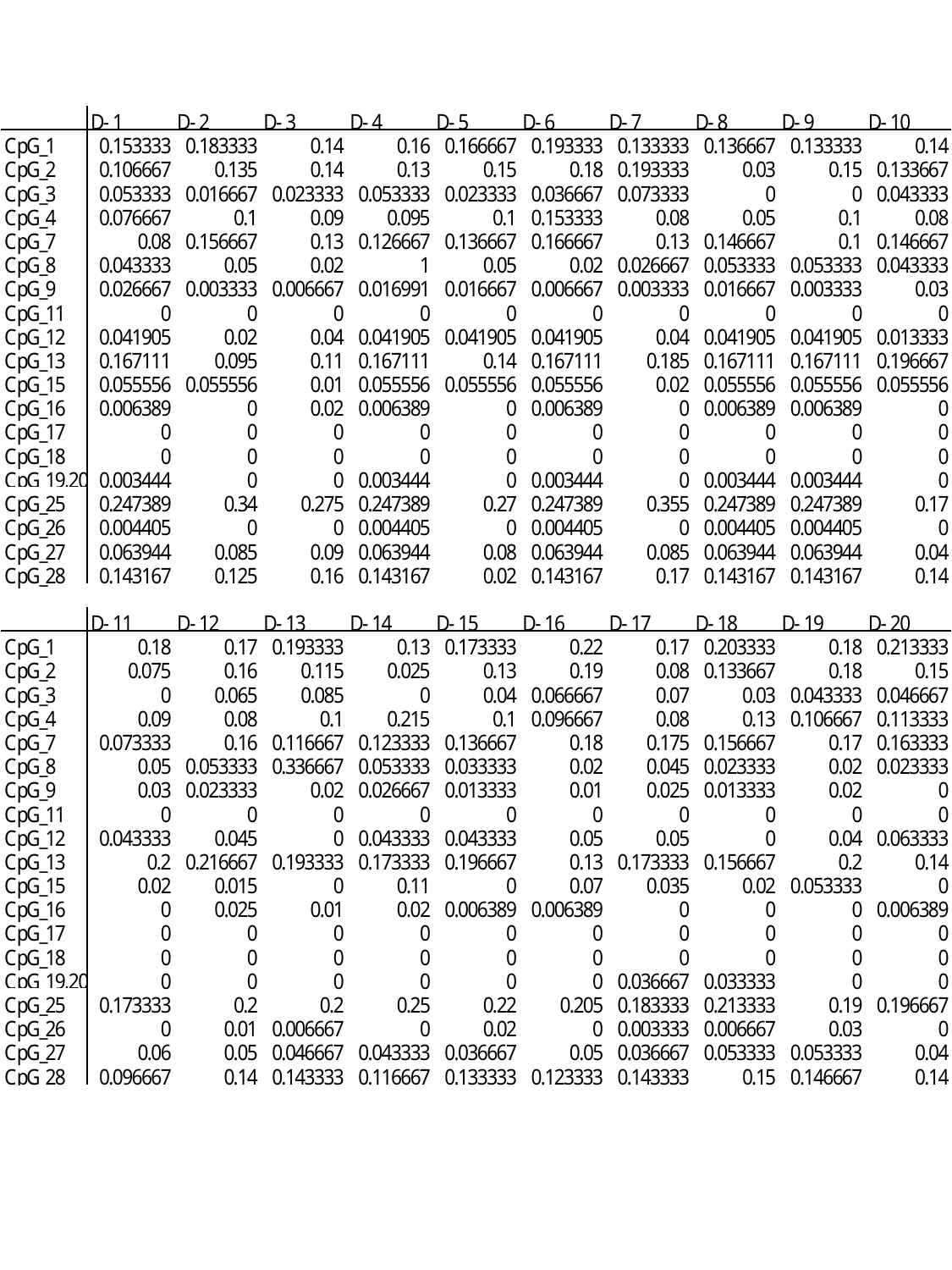

Supplement: Data S2 — Raw data from MassArray analysis of CpG IV. (PPT) [file pone.0023881.s002.ppt]
