# Supplementary figures and images for: DNA Methylation Profiles of the Brain-Derived Neurotrophic Factor (BDNF) Gene as a Potent Diagnostic Biomarker in Major Depression
Source: PLoS One. 2011 Aug 30;6(8):e23881. doi: 10.1371/journal.pone.0023881 (PMC3166055; doi:10.1371/journal.pone.0023881)

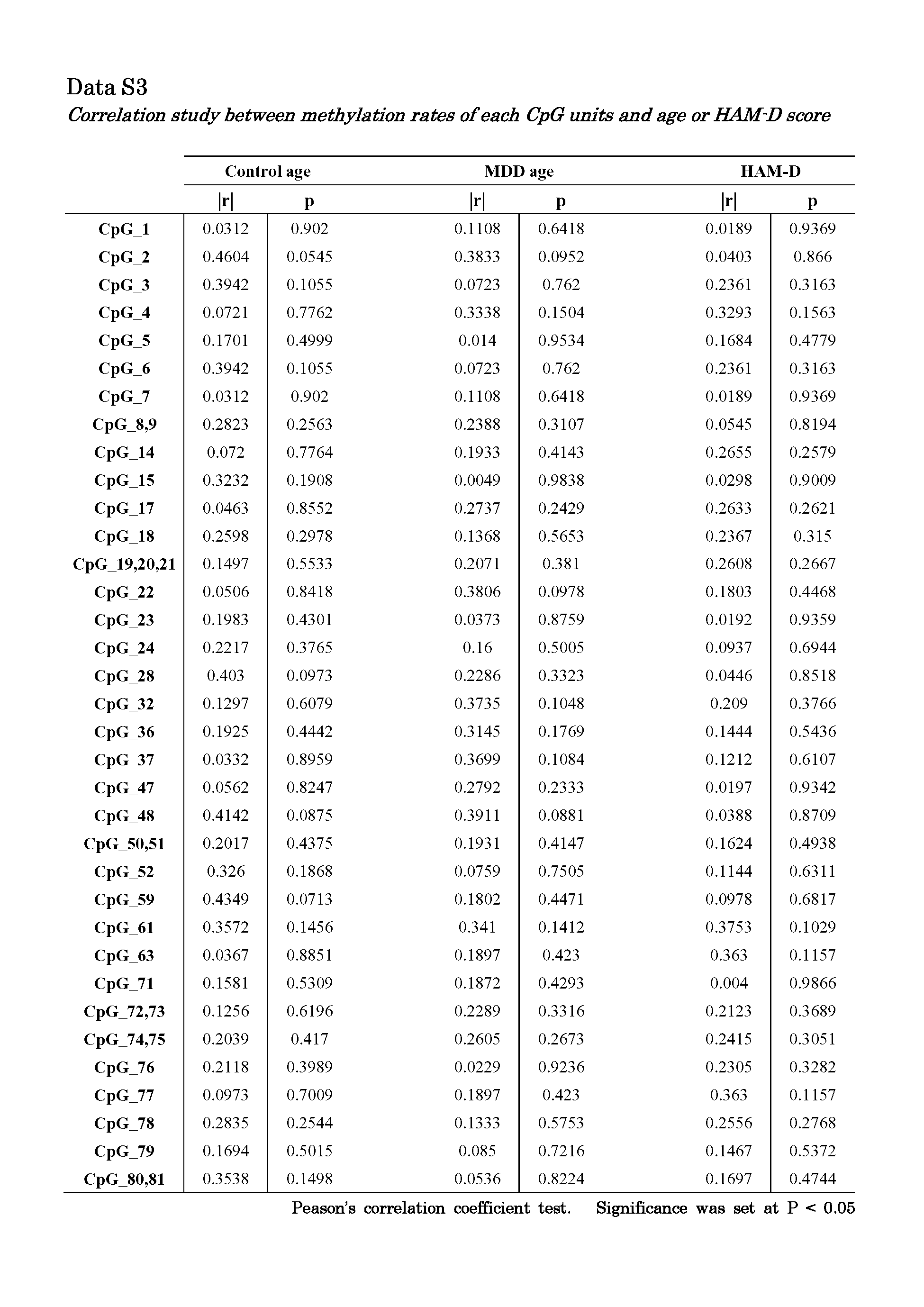

Supplement: Data S3 — Correlation study between methylation rates of each CpG units and age or HAM-D score. (TIF) [file pone.0023881.s003.tif]
